# Emapalumab in IFN-γ-Driven Hyperinflammatory Disorders: Mechanisms, Clinical Evidence, and Emerging Therapeutic Applications

**DOI:** 10.3390/cimb48100981

**Published:** 2026-09-24

**Authors:** Yayi Qian, Xiaojun Xu

**Affiliations:** Department of Hematology & Oncology, Children′s Hospital, Zhejiang University School of Medicine, Hangzhou 310003, China; qianyayi@zju.edu.cn

**Keywords:** emapalumab, interferon-γ, hemophagocytic lymphohistiocytosis, macrophage activation syndrome, hyperinflammation, clinical trials

## Abstract

Hyperinflammatory disorders are severe clinical syndromes characterized by uncontrolled immune activation, sustained release of inflammatory mediators, and multiorgan injury. Hemophagocytic lymphohistiocytosis (HLH) and macrophage activation syndrome (MAS) are two representative hyperinflammatory syndromes. Growing evidence has identified interferon gamma (IFN-γ) as an important mediator, contributing to persistent immune activation and amplification of inflammatory responses. Emapalumab is a fully human, high-affinity monoclonal antibody targeting IFN-γ that exerts its therapeutic effects by selectively neutralizing IFN-γ and suppressing its downstream inflammatory activity. Clinical studies have established the therapeutic utility of emapalumab in primary HLH, while its potential applications are increasingly being explored in Still’s disease-associated MAS, secondary HLH, chimeric antigen receptor (CAR) T-cell therapy-associated hyperinflammatory states, and hematopoietic stem cell transplantation-related settings. This review summarizes the biological basis of IFN-γ-driven hyperinflammation, the molecular mechanism of action of emapalumab, and current clinical evidence for its use in HLH, MAS, and other hyperinflammatory conditions, while discussing the limitations of existing evidence and priorities for future research.

## 1. Introduction

Hyperinflammatory disorders comprise a group of severe conditions characterized by uncontrolled immune activation and excessive release of inflammatory mediators, which can lead to cytopenias, coagulopathy, tissue injury, and multiorgan dysfunction. Hemophagocytic lymphohistiocytosis (HLH) and macrophage activation syndrome (MAS) are among the most representative hyperinflammatory syndromes [1]. HLH may arise from inherited defects in immune function or occur secondary to infections, malignancies, rheumatic diseases, and immune-based therapies [2]. Persistent immune activation and dysregulated cytokine production can rapidly result in severe organ damage and death [3]. MAS occurs most commonly in the context of Still’s disease, including systemic juvenile idiopathic arthritis (sJIA) and adult-onset Still’s disease (AOSD), and is characterized by an acute escalation of systemic inflammation with multiorgan involvement [1,4].

Despite advances in therapeutic strategies, refractory, recurrent, or progressive HLH and MAS remain major therapeutic challenges [5].

Advances in our understanding of the immunopathogenesis of hyperinflammatory disorders have facilitated the development of targeted therapies. In HLH and MAS, sustained interferon gamma (IFN-γ) signaling has been implicated in the amplification of inflammatory responses and tissue injury [4,6,7]. These findings provide a strong biological rationale for therapeutically targeting the IFN-γ pathway. Emapalumab is a fully human monoclonal antibody that specifically binds and neutralizes IFN-γ, thereby inhibiting its biological activity [8]. In 2018, the U.S. Food and Drug Administration approved emapalumab for the treatment of pediatric and adult patients with primary HLH (pHLH) who have refractory, recurrent, or progressive disease or are intolerant of conventional HLH therapy [9]. Since then, clinical studies of emapalumab have expanded to MAS and other hyperinflammatory conditions, extending investigation of IFN-γ blockade beyond pHLH [10]. This review summarizes the role of IFN-γ in hyperinflammatory disorders and the mechanism of action of emapalumab, with a particular focus on emerging clinical evidence for its use in HLH, MAS, and other hyperinflammatory conditions.

## 2. Biological Role of IFN-γ in Hyperinflammation

IFN-γ, the sole type II interferon, is produced predominantly by activated T cells and natural killer (NK) cells and plays an important role in host defense against infection, macrophage activation, and antigen presentation [11,12]. Upon binding to the IFN-γ receptor (IFNGR), IFN-γ activates the Janus kinase 1/2 (JAK1/JAK2)–signal transducer and activator of transcription 1 (STAT1) signaling pathway and induces the transcription of IFN-γ-responsive genes [13,14,15]. In macrophages, this signaling enhances antimicrobial activity and antigen presentation and promotes the production of inflammatory mediators and chemokines, including C-X-C motif chemokine ligand 9 (CXCL9) and CXCL10, thereby facilitating the recruitment of activated lymphocytes and coordinating interactions between lymphoid and myeloid cells [16,17,18]. Under physiological conditions, IFN-γ production is transient and tightly regulated, allowing these responses to promote pathogen clearance and effective cell-mediated immunity while limiting excessive inflammation and tissue injury [19].

In HLH and MAS, persistent immune activation disrupts this tightly controlled response and shifts IFN-γ from a protective immune mediator toward a major effector of pathological inflammation [20]. Sustained activation of T and NK cells leads to excessive IFN-γ production, which further promotes macrophage activation, antigen presentation, and the production of proinflammatory cytokines and chemokines [7,21]. These mediators recruit and activate additional immune cells, providing further sources of IFN-γ and establishing a self-amplifying inflammatory circuit between lymphoid and myeloid compartments [22]. Persistent activation of this circuit contributes to systemic inflammation, hemophagocytosis, tissue injury, cytopenias, coagulopathy, and ultimately multiorgan dysfunction [7,23]. Such IFN-γ-driven inflammation can arise from heterogeneous upstream triggers, including genetic defects in lymphocyte cytotoxicity, persistent infection, malignancy, rheumatic disease, and immune-based therapies such as chimeric antigen receptor (CAR) T-cell therapy [24,25]. Importantly, IFN-γ acts within a broader and context-dependent cytokine network involving interleukin-1 (IL-1), IL-6, IL-18, tumor necrosis factor-alpha (TNF-α), and other mediators [26,27,28]. The relative contribution of these pathways differs among hyperinflammatory syndromes and may also vary between individual patients within the same disease [29]. For example, IL-18 can promote IFN-γ production and is particularly prominent in Still’s disease-associated MAS [26,28], whereas IL-6 has a major pathogenic and therapeutic role in CAR T-cell-associated cytokine release syndrome (CRS) [30]. In other settings, several cytokine pathways may operate simultaneously or sequentially. Therefore, the detection of elevated IFN-γ alone should not be interpreted as evidence that a disease is functionally dependent on IFN-γ signaling or that selective IFN-γ blockade will necessarily result in clinical benefit [4].

This distinction between IFN-γ elevation and IFN-γ dependence is important when considering emapalumab therapy. IFN-γ elevation indicates activation of the pathway, whereas IFN-γ dependence implies that IFN-γ makes a sufficiently dominant causal contribution to the inflammatory phenotype such that its neutralization results in meaningful disease control. These biological differences may partly explain the heterogeneous clinical responses to IFN-γ blockade observed across pHLH, MAS, secondary HLH (sHLH), and CAR T-cell-associated hyperinflammatory syndromes. Consequently, identifying functional IFN-γ dependence, rather than IFN-γ elevation alone, represents an important challenge for biomarker-guided use of emapalumab [31] (Figure 1).

## 3. Molecular Mechanism of Action of Emapalumab

Emapalumab is a fully human IgG1 monoclonal antibody that binds IFN-γ with high affinity and selectively neutralizes its biological activity through a noncompetitive mechanism [9]. Under physiological conditions, IFN-γ signaling is initiated by its binding to IFNGR1, followed by the recruitment of IFNGR2 to form a functional receptor complex. By binding to IFN-γ, emapalumab interferes with the formation of this signaling-competent complex, thereby inhibiting downstream JAK1/JAK2–STAT1 activation and the expression of IFN-γ-responsive genes, including the chemokines CXCL9 and CXCL10 [8,32].

This mechanism distinguishes emapalumab from conventional HLH-directed therapies such as glucocorticoids and etoposide, which reduce immune activation through broader immunosuppressive or cytotoxic effects [33]. In contrast, emapalumab selectively neutralizes IFN-γ, providing a targeted approach to modulating IFN-γ-mediated inflammation in disease settings in which this pathway contributes substantially to pathogenesis (Figure 2).

## 4. Emapalumab in HLH

Emapalumab is the first monoclonal antibody targeting the IFN-γ pathway to be approved for the treatment of HLH. Its clinical development initially focused on pHLH and has subsequently expanded to sHLH and hematopoietic stem cell transplantation (HSCT)-related settings (Table 1) [2].

### 4.1. Primary HLH

The rationale for IFN-γ blockade in pHLH was initially established in preclinical models of inherited HLH, in which IFN-γ neutralization ameliorated disease manifestations, reduced tissue inflammation, and improved survival [46]. Subsequent pharmacokinetic/pharmacodynamic (PK/PD) analyses of emapalumab further supported this therapeutic strategy [8]. Because emapalumab forms stable drug–target complexes with IFN-γ, total IFN-γ measured during treatment comprises both free and drug-bound cytokine and therefore does not directly reflect residual IFN-γ bioactivity. In contrast, CXCL9 production is highly dependent on IFN-γ–IFNGR signaling and can serve as a pharmacodynamic marker of pathway activity [47]. PK/PD analyses showed that increasing emapalumab exposure was associated with decreasing CXCL9 levels, while lower CXCL9 levels were associated with a greater probability of clinical response, further supporting the pharmacodynamic relationship between IFN-γ pathway inhibition and clinical improvement [48,49]. In addition, emapalumab pharmacokinetics are influenced by IFN-γ levels, with higher total IFN-γ concentrations associated with increased drug clearance. This relationship provides a pharmacological basis for individualized dose adjustment during treatment [50,51].

The efficacy and safety of emapalumab in pHLH were evaluated in an open-label, single-arm phase II–III study (NCT01818492), in which emapalumab was administered in combination with dexamethasone. Among 34 enrolled patients, 27 had previously received HLH-directed therapy. In this previously treated population, the overall response rate was 63%, significantly exceeding the prespecified null hypothesis of 40% (*p* = 0.02), with complete and partial responses achieved in 26% and 30% of patients, respectively. At the last observation, 74% of previously treated patients were alive, with an estimated 12-month overall survival probability of 73.4% [8]. These findings established the clinical activity of selective IFN-γ blockade in patients with previously treated pHLH. The REAL-HLH study subsequently extended these findings to routine clinical practice. This multicenter retrospective study included 46 patients with pHLH treated with emapalumab across 33 hospitals in the United States. CXCL9 normalized in 72.7% (24/33) of evaluable patients, accompanied by improvements in several other HLH-related laboratory parameters. Overall survival at the end of follow-up was 73.9%, and the estimated probability of survival at 12 months after emapalumab initiation was 73.1% [34]. Collectively, clinical trial and real-world evidence support the use of emapalumab to control hyperinflammation in patients with refractory, recurrent, or progressive pHLH.

### 4.2. Secondary HLH

sHLH can be triggered by a broad range of conditions, including infections, malignancies, rheumatic diseases, and immune-based therapies. Despite considerable heterogeneity in the underlying triggers, persistent activation of T and NK cells and excessive IFN-γ production are prominent features of hyperinflammation in a subset of patients with sHLH. International recommendations for the management of adult HLH emphasize that the underlying trigger, including infection or malignancy, should be treated concurrently with the hyperinflammatory state [3]. Accordingly, emapalumab has been used in sHLH primarily to control IFN-γ-driven hyperinflammation as an adjunct to therapies directed at the underlying disease.

#### 4.2.1. EBV-Associated HLH

Epstein–Barr virus (EBV) is one of the most common infectious triggers of sHLH. Persistent viral stimulation can lead to aberrant activation of T and NK cells and excessive production of inflammatory cytokines. Recent single-cell transcriptomic studies have revealed distinct immune activation signatures in EBV-HLH, including T/NK-cell activation and inflammatory pathway remodeling [52,53].

Current clinical evidence remains limited and is derived mainly from retrospective studies and small case series. In a 2026 single-center retrospective study of 10 pediatric patients with HLH treated with emapalumab-based combination therapy, seven had refractory disease, two had relapsed disease, and six had EBV-associated HLH. Five of the 10 patients achieved remission. Improvements were observed in fever, hypofibrinogenemia, and neutropenia, with response rates of 6/6, 5/5, and 8/9, respectively [35]. In a separate case series of four pediatric patients with refractory EBV-HLH, three achieved disease control after emapalumab was added to HLH-94- or L-DEP-based therapy, accompanied by reductions in inflammatory markers and improvements in blood counts, liver function, and EBV viral load. The remaining patient had severe multiorgan dysfunction, failed to achieve disease control, and discontinued treatment [54]. An additional pediatric case report described hematologic remission after emapalumab in a child with relapsed/refractory EBV-HLH, followed by successful allogeneic hematopoietic cell transplantation [55].

Evidence in adult EBV-HLH is even more limited. A report of three adult patients treated with low-dose emapalumab in combination with chemotherapy showed rapid control of disease manifestations and improvement in hematologic parameters, with all three subsequently proceeding to allogeneic HSCT [56]. However, these responses should be interpreted cautiously, as improvement in fever, ferritin, CXCL9, or other inflammatory parameters does not necessarily indicate control of the underlying EBV-driven disease. Emapalumab may serve as an adjunctive therapy for rapid control of life-threatening hyperinflammation and stabilization of patients with refractory EBV-HLH, particularly when used alongside treatment directed at the underlying EBV-associated disease. In this setting, emapalumab should not be regarded as a substitute for definitive treatment of the underlying trigger. The current evidence base is limited to retrospective cohorts and small case series or case reports, and the optimal timing, combination strategy, and long-term clinical benefit remain to be defined in larger prospective studies.

#### 4.2.2. Malignancy-Associated HLH

Malignancy-associated HLH (M-HLH) is a severe subtype of sHLH associated with particularly dismal outcomes; it occurs most frequently in the setting of lymphoma, leukemia, and other hematologic malignancies [57]. Hyperinflammation in M-HLH may result from persistent tumor-associated immune stimulation and dysregulated cytokine production [58,59], and may be further exacerbated by concurrent infections and treatment-related immune dysregulation.

Clinical evidence for emapalumab in M-HLH remains limited and is derived primarily from retrospective and real-world studies. In a retrospective study of 15 adults with M-HLH treated with emapalumab as salvage therapy, the median overall survival was only 18 days from M-HLH diagnosis and 4 days from emapalumab initiation, reflecting the severe clinical condition of this population. Seven patients died or entered hospice care within 72 h of the first dose. Among the eight patients evaluable for biomarker response, seven (88%) achieved a ferritin response, with a median reduction of 64%; however, overall survival remained poor [36]. The REAL-HLH study subsequently provided additional real-world evidence in 17 patients with M-HLH. Emapalumab was initiated predominantly for refractory or progressive disease, and most patients received other HLH-directed therapies before or concurrently with emapalumab. Several HLH-related laboratory parameters improved or remained within the normal range during emapalumab-containing treatment, with CXCL9 normalizing or remaining normal in 7/8 (87.5%) evaluable patients. Nevertheless, overall survival at the end of follow-up was 23.5%, and the estimated 12-month survival probability from emapalumab initiation was 22.1% [37]. More recent evidence has further implicated IFN-γ in M-HLH-associated organ injury. In a retrospective cohort of 53 patients with NK/T-cell lymphoma, all 18 patients who developed HLH had hepatic injury, and serum IFN-γ levels were positively correlated with GGT, AST, and direct bilirubin levels. Two patients with NK/T-cell lymphoma-associated HLH and severe hepatic injury showed rapid improvement in liver function following treatment with emapalumab in combination with chemotherapy [60]. Collectively, these findings suggest that emapalumab-containing regimens may suppress IFN-γ-driven hyperinflammation and improve selected inflammatory or organ injury-related parameters in M-HLH. However, the persistently poor survival observed despite biochemical improvement indicates that suppression of hyperinflammation alone may be insufficient to achieve durable disease control. Outcomes remain strongly influenced by the underlying malignancy, infection, organ dysfunction, and treatment-related complications. Because emapalumab does not directly target the underlying malignancy, it is best positioned as an adjunctive strategy to stabilize patients and facilitate malignancy-directed therapy whenever clinically feasible, rather than as a definitive treatment for M-HLH.

#### 4.2.3. CAR T-Cell-Associated CRS and HLH-like Syndrome

CAR T-cell therapy uses genetically engineered T cells to recognize and eliminate tumor cells and has become an established treatment for several hematologic malignancies [61,62]. CRS and immune effector cell-associated HLH-like syndrome (IEC-HS) are related but distinct hyperinflammatory toxicities following CAR T-cell therapy. CRS is an acute systemic inflammatory syndrome characterized primarily by fever, with severity determined by the degree of hypotension and hypoxia. IEC-HS, in contrast, is an HLH-like syndrome characterized by marked hyperferritinemia together with features such as cytopenias, coagulopathy or hypofibrinogenemia, hepatic dysfunction, and other manifestations of macrophage activation [63,64]. The inflammatory pathways underlying these syndromes also differ. CRS involves broad cytokine release following CAR T-cell and myeloid-cell activation, with IL-6 representing an important therapeutic target; IFN-γ pathway activation may occur but constitutes only one component of this broader inflammatory network. In IEC-HS, sustained lymphoid–myeloid activation and the HLH-like phenotype may be associated with a more prominent IFN-γ signature in selected patients, providing a biological rationale for IFN-γ blockade with emapalumab in refractory disease. However, this mechanistic rationale should not be equated with established clinical efficacy. Evidence for emapalumab in severe CRS is currently limited to small retrospective studies, while evidence in IEC-HS is even more restricted and consists mainly of case reports and very small case series.

Preclinical studies provide a mechanistic rationale for targeting IFN-γ in CAR T-cell-associated hyperinflammation. IFN-γ blockade or genetic deletion reduced macrophage activation and macrophage-derived inflammatory cytokines and chemokines without compromising CAR T-cell cytotoxicity against hematologic malignancies; CAR T-cell proliferation was preserved or enhanced in experimental models [65]. Early clinical reports subsequently described the use of emapalumab in severe CAR T-cell–associated CRS refractory to conventional anti-inflammatory therapy, providing initial clinical experience with this strategy [66]. Subsequently, a 2026 retrospective study evaluated 38 pediatric patients with CAR T-cell-associated CRS who had an inadequate response to glucocorticoids and/or tocilizumab, 37 (97%) of whom had grade ≥ 3 CRS. Following emapalumab treatment, body temperature and multiple inflammatory cytokines decreased markedly, with measured circulating IFN-γ concentrations decreasing from 21,984.11 to 674.87 pg/mL. Meanwhile, both the absolute number of circulating CAR T cells and their proportion among CD3^+^ T cells increased, with no apparent evidence of impaired short-term CAR T-cell expansion. The 6-month event-free survival and overall survival rates were 76.9% and 80.1%, respectively. These findings provide preliminary clinical evidence supporting further investigation of emapalumab as a salvage strategy for refractory high-grade CRS [44]. A separate case series of six children and adolescents with refractory CRS similarly reported reductions in inflammatory markers and improvement in clinical status following emapalumab treatment. Antitumor responses to CAR T-cell therapy were maintained in evaluable patients, providing additional preliminary evidence for the feasibility of IFN-γ blockade in CAR T-cell-associated hyperinflammation [43].

For patients who develop IEC-HS or other HLH/MAS-like hyperinflammatory manifestations following CAR T-cell therapy, however, the available evidence remains largely limited to individual case reports. Early reports described control of CAR T-cell-associated MAS/HLH after the addition of emapalumab to glucocorticoids and anakinra [67]. Subsequent cases of refractory IEC-HS in adults have similarly reported improvements in inflammatory markers and organ function following emapalumab after failure of multiple anti-inflammatory therapies [68]. More recently, a patient with persistent IEC-HS characterized by prolonged transfusion-dependent cytopenias and elevated CXCL9 was treated with emapalumab in combination with eltrombopag. IFN-γ and CXCL9 were subsequently suppressed, and the patient ultimately achieved transfusion independence; notably, stringent complete remission with minimal residual disease (MRD) negativity was maintained 2 years after CAR T-cell therapy [69]. Collectively, these cases provide preliminary support for emapalumab as a mechanism-based therapeutic option for refractory IEC-HS. However, its optimal timing and potential effects on long-term CAR T-cell persistence and antitumor efficacy require further investigation.

### 4.3. Therapeutic Role Before and After HSCT

Hematopoietic stem cell transplantation (HSCT) remains the mainstay of treatment for achieving long-term disease control and potential cure in patients with pHLH, particularly those with defined genetic defects or refractory or recurrent disease [33,70]. Because active HLH and a high inflammatory burden before transplantation may adversely affect transplant outcomes, adequate control of hyperinflammation before HSCT is an important component of treatment [71,72]. In this setting, emapalumab may facilitate disease control by suppressing IFN-γ-mediated inflammation and serve as a bridge to HSCT in selected patients.

In the pivotal phase II–III study, 19 of 27 (70%) previously treated patients proceeded to HSCT following emapalumab therapy, with an estimated 12-month post-HSCT survival probability of 89.5% [8]. Real-world data from the REAL-HLH study provide further evidence for the use of emapalumab before transplantation. Among 46 patients with pHLH, 42 were considered eligible for transplantation, of whom 31 (73.8%) underwent HSCT; pretransplant survival was 90.5%, and the estimated 12-month post-HSCT survival probability was 73.7% [34]. A subsequent transplant-focused analysis of the REAL-HLH cohort provided more detailed post-transplant outcomes in 35 patients who underwent their first HSCT after emapalumab treatment, of whom 29 (82.9%) had pHLH. Among 30 patients with evaluable engraftment data, 24 (80%) achieved engraftment without graft failure, second transplantation, or stem-cell boost, with a median time to engraftment of 16.1 days. Mixed chimerism occurred in 13 of 35 patients (37.1%), and the 12-month post-HSCT survival probability was 73.4% [39]. Beyond its role in bridging patients to transplantation, recent studies have investigated whether emapalumab exposure before HSCT may also influence donor chimerism and longer-term transplant outcomes. In a retrospective study of 50 pediatric patients with HLH undergoing reduced-intensity conditioning HSCT, 22 received emapalumab within 21 days before conditioning. Emapalumab exposure was associated with lower rates of mixed chimerism (48% vs. 77%, *p* = 0.03) and severe mixed chimerism (5% vs. 38%, *p* < 0.01), as well as higher 5-year intervention-free survival (73% vs. 43%, *p* = 0.03), whereas overall survival did not differ significantly (82% vs. 71%) [40].

The potential application of emapalumab has also been explored in immune-mediated graft failure after HSCT. A multicenter retrospective case series reported the use of emapalumab in patients with suspected immune-mediated graft failure, providing preliminary evidence for targeting IFN-γ in this setting [73]. More recently, a patient with familial HLH type 3 (FHL3) developed early peri-graft HLH reactivation after haploidentical HSCT, characterized by increased IFN-γ, ferritin, and soluble IL-2 receptor levels and an imminent risk of immune-mediated graft failure. Emapalumab was added to corticosteroids and anakinra, followed by progressive resolution of hyperinflammation and eventual achievement of complete donor chimerism [74]. This case suggests that, in patients with pHLH and persistent IFN-γ-driven inflammation during the peri-transplant period, emapalumab may have a potential role in controlling HLH reactivation and supporting successful engraftment. However, evidence for this application remains limited to individual case reports. Overall, the potential role of emapalumab in the HSCT setting has expanded from pretransplant disease control and bridging to transplantation to the modulation of donor chimerism and transplant-related immune complications. These emerging applications, however, require validation in prospective studies.

## 5. Emapalumab in MAS

As the role of IFN-γ in the hyperinflammatory response associated with Still’s disease-associated MAS has become increasingly recognized, selective IFN-γ blockade has emerged as a potential therapeutic strategy for refractory MAS [75,76]. The clinical efficacy of emapalumab in Still’s disease-associated MAS was first evaluated in a prospective, open-label study involving 14 patients with an inadequate response to high-dose glucocorticoids. By week 8, 13 patients (93%) achieved MAS remission, with a median time to remission of 25 days, accompanied by a rapid decline in the IFN-γ-related biomarker CXCL9 [41]. Subsequent pharmacokinetic/pharmacodynamic (PK/PD) analyses showed estimated reductions of 98.3%, 87.0%, and 99.6% in the production rates of CXCL9, soluble IL-2 receptor α, and ferritin, respectively, further supporting the pharmacodynamic relationship between IFN-γ pathway inhibition and improvement in MAS-associated inflammation. Viral infections or positive virologic findings were observed during treatment, highlighting the importance of infection surveillance during IFN-γ blockade [31].

Evidence has also begun to extend to other rheumatic disease-associated HLH/MAS. In the REAL-HLH multicenter study of 15 patients with rheumatic disease-associated HLH, CXCL9 normalized or remained within the normal range in 9 of 11 evaluable patients (81.8%), and the 12-month survival probability from emapalumab initiation was 86.7% [38]. A 2025 retrospective series evaluated low-dose emapalumab in nine patients with refractory MAS associated with AOSD or systemic lupus erythematosus (SLE), including four patients with SLE. Overall response rates increased from 66.7% at week 1 to 88.9% at week 8, and eight of nine patients achieved complete remission by the end of follow-up [42]. Beyond these cohort studies, individual case reports have extended the clinical experience with emapalumab to severe and treatment-refractory MAS. A 15-year-old patient with severe treatment-refractory MAS achieved sustained clinical and laboratory remission with emapalumab after inadequate responses to high-dose glucocorticoids, anakinra, and cyclosporine [77]. In another report, an adult with refractory SLE-associated HLH/MAS maintained remission during prolonged emapalumab therapy after failure of previous treatments and responded again to emapalumab following disease recurrence [78]. Although these observations suggest a potential role for emapalumab in patients with refractory disease after multiple lines of therapy, its long-term efficacy and optimal treatment duration remain to be established.

## 6. Emapalumab Beyond HLH and MAS

Thrombotic microangiopathy (TMA) can occur concomitantly with hyperinflammatory conditions such as HLH and MAS and is characterized by endothelial injury, dysregulated complement activation, and microvascular thrombosis. Because hyperinflammation and complement-mediated endothelial injury may coexist, combination strategies targeting these distinct pathogenic pathways have attracted increasing interest [79,80,81].

A retrospective study included 23 patients with refractory HLH, 16 (69.6%) of whom met criteria for high-risk TMA. Ten patients received the complement inhibitor eculizumab for TMA while undergoing HLH-directed therapy. Notably, all patients treated with eculizumab in combination with emapalumab achieved complete resolution of TMA and survived [45]. These observations provide preliminary evidence that simultaneous inhibition of IFN-γ and complement may be beneficial in selected patients with HLH complicated by TMA. A similar combination strategy has also been explored in MAS complicated by TMA. In a 2025 case report, a 41-year-old patient with MAS and TMA experienced continued disease progression despite multiple immunomodulatory therapies and achieved only disease stabilization after 4 weeks of eculizumab. Following the addition of emapalumab, marked improvements were observed in lactate dehydrogenase, ferritin, and platelet counts, together with recovery of fibrinogen and complement levels [82]. Although the available evidence is currently limited to a small retrospective study and individual case reports, these observations suggest that combined blockade of IFN-γ and complement may represent a therapeutic strategy worth further investigation in patients with concomitant IFN-γ-driven hyperinflammation and complement-mediated microvascular injury.

## 7. Safety

Available clinical evidence indicates that emapalumab is generally well tolerated, with infections representing the major safety concern during treatment. IFN-γ plays an important role in host defense against intracellular pathogens, and selective IFN-γ blockade may therefore increase susceptibility to certain infections. In the phase II–III study of pHLH, serious infections occurred in 10 of 34 patients treated with emapalumab, including one case of disseminated histoplasmosis that led to treatment discontinuation. Infusion-related reactions occurred in approximately 27% of patients but were not severe, and no apparent emapalumab-related myelosuppression or organ toxicity was observed [8]. Viral infection-related events were also reported in studies of Still’s disease-associated MAS, including cytomegalovirus (CMV) reactivation or CMV test positivity [41,83]. Importantly, patients with HLH and MAS have substantial underlying immune dysregulation and frequently receive concomitant glucocorticoids, etoposide, or other immunosuppressive therapies, making it difficult to attribute infectious complications solely to emapalumab. Given the role of IFN-γ in antimicrobial immunity, screening for and close monitoring of infections are warranted before and during treatment, with particular attention to mycobacterial infections, histoplasmosis, and herpesvirus reactivation [8]. As the clinical use of emapalumab expands beyond pHLH to MAS, sHLH, and other hyperinflammatory conditions, larger studies with longer follow-up will be needed to better characterize its safety profile across different disease settings.

## 8. Discussion

As a cytokine-targeted therapy, emapalumab provides a mechanism-based therapeutic strategy for hyperinflammation through direct neutralization of IFN-γ. Its clinical role, however, should be interpreted according to the specific disease context and strength of available evidence. Whereas its role is best established in pHLH, prospective clinical evidence is also available for Still’s disease-associated MAS; in contrast, evidence in many secondary or emerging settings is derived largely from retrospective studies, small case series, or case reports. Because hyperinflammation is often driven by multiple interacting cytokine pathways, IFN-γ blockade alone may be insufficient in patients whose disease is not functionally dependent on IFN-γ. Moreover, emapalumab primarily suppresses the inflammatory response but does not eliminate underlying disease drivers such as persistent infection or malignancy. This limitation is particularly evident in M-HLH, where improvement in inflammatory parameters has been observed despite poor overall survival, suggesting that improvement in inflammatory biomarkers does not necessarily translate into durable disease control. In these evidence-limited and mechanistically complex settings, emapalumab may therefore be more appropriately considered an adjunctive, salvage, or bridging strategy rather than a substitute for treatment directed at the underlying disease. The potential benefits of targeted inflammatory control must also be balanced against the infectious risks associated with IFN-γ blockade.

This heterogeneity in disease mechanisms and treatment responses further highlights the need for improved biomarker-guided patient selection. CXCL9 is a relatively proximal pharmacodynamic marker of IFN-γ–IFNGR signaling and may more accurately reflect pathway activity than total circulating IFN-γ during emapalumab treatment; however, elevated CXCL9 alone does not establish IFN-γ dependence. No prospectively validated biomarker threshold currently identifies patients most likely to benefit from IFN-γ blockade. Future patient-selection strategies may therefore require the integration of CXCL9 with other inflammatory biomarkers, clinical phenotype, underlying disease context, and dynamic treatment responses to better distinguish IFN-γ pathway activation from therapeutically relevant functional IFN-γ dependence.

## 9. Conclusions

Emapalumab selectively neutralizes IFN-γ and represents a mechanism-based targeted therapy for IFN-γ-driven hyperinflammation. However, the strength of clinical evidence varies considerably across disease settings, with the strongest evidence in pHLH and prospective evidence in Still’s disease-associated MAS, while its use in other hyperinflammatory conditions remains investigational. Future studies should better define the disease contexts and patient profiles most likely to benefit from IFN-γ blockade.

## Figures and Tables

**Figure 1:**
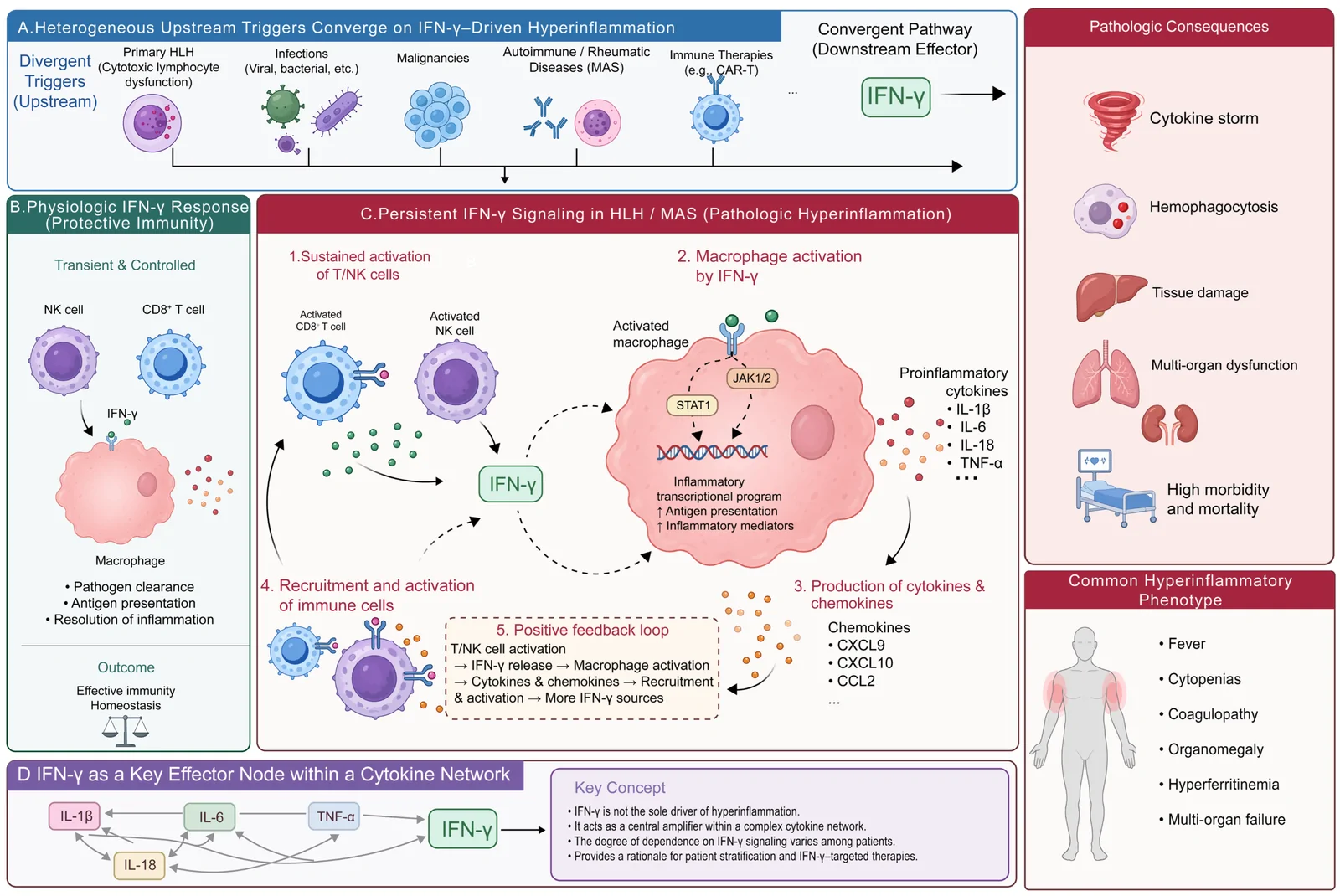
Physiological and pathological roles of IFN-γ in hyperinflammatory disorders. (**A**) Diverse triggers converge on IFN-γ-associated inflammatory pathways. (**B**) Physiological IFN-γ promotes antimicrobial activity and antigen presentation. (**C**) In HLH and MAS, sustained T/NK-cell activation drives persistent IFN-γ signaling, macrophage activation, and a self-amplifying inflammatory loop. (**D**) IFN-γ acts within a broader cytokine network, with variable contributions across disease settings. Abbreviations: IFN-γ, interferon gamma; JAK, Janus kinase; STAT1, signal transducer and activator of transcription 1; CAR, chimeric antigen receptor; CXCL9, C-X-C motif chemokine ligand 9; CXCL10, C-X-C motif chemokine ligand 10; IL-1, interleukin-1; IL-6, interleukin-6; IL-18, interleukin-18; TNF-α, tumor necrosis factor-alpha; CCL2, C-C motif chemokine ligand 2; HLH, hemophagocytic lymphohistiocytosis; MAS, macrophage activation syndrome; NK, natural killer.

**Figure 2:**
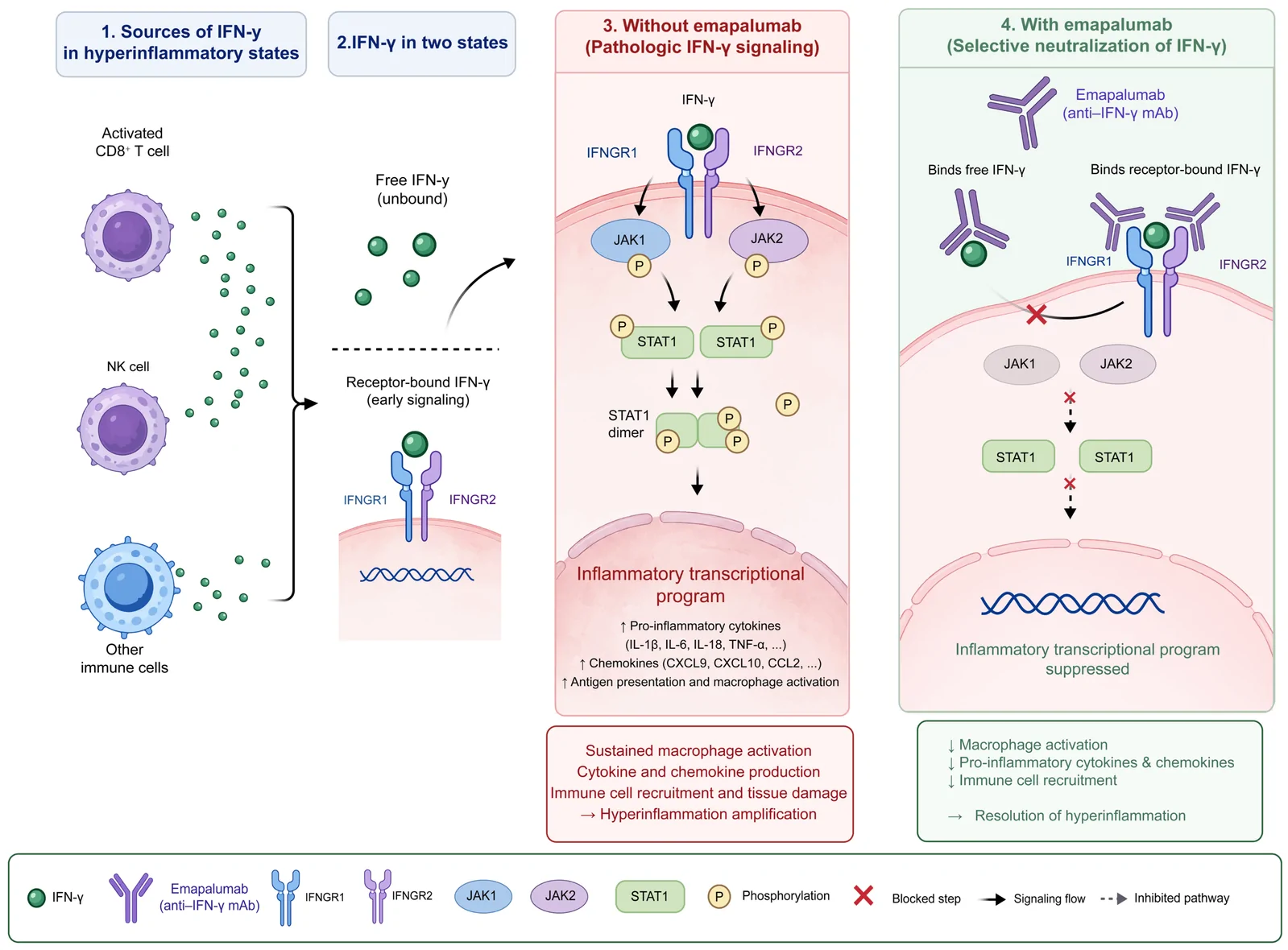
Molecular mechanism of action of emapalumab in IFN-γ-driven hyperinflammation. IFN-γ activates IFNGR–JAK1/JAK2–STAT1 signaling and downstream inflammatory responses. Emapalumab selectively binds and neutralizes IFN-γ, preventing productive receptor signaling and suppressing downstream pathway activation. This targeted blockade attenuates IFN-γ-dependent inflammation and the inflammatory interaction between activated lymphocytes and macrophages. Abbreviations: IFN-γ, interferon gamma; IFNGR, interferon-gamma receptor; JAK, Janus kinase; STAT1, signal transducer and activator of transcription 1; IL-1β, interleukin-1 beta; IL-6, interleukin-6; IL-18, interleukin-18; TNF-α, tumor necrosis factor-alpha; CXCL9, C-X-C motif chemokine ligand 9; CXCL10, C-X-C motif chemokine ligand 10; CCL2, C-C motif chemokine ligand 2; NK, natural killer.

**Table 1:** Clinical evidence for emapalumab in hyperinflammatory disorders.

Disease	Population/Subtype	Study/Design	n	Emapalumab Regimen	Main Outcome	Major Safety Considerations	Ref.
**pHLH**	Previously treated	Phase II–III, open-label	27	Emapalumab + dexamethasone	ORR 63%; CR 26%; 70% bridged to HSCT	Serious infections; infusion reactions; histoplasmosis	[8]
	Real-world	Multicenter retrospective	46	Emapalumab-containing treatment in routine practice	CXCL9 normalization 72.7%; 12-month OS 73.1%	Infection	[34]
**sHLH**	Pediatric HLH (including 6 EBV-HLH)	Single-center retrospective	10	Emapalumab-based combination therapy ± conventional HLH treatment/ruxolitinib	Remission 50%	Infections	[35]
	Malignancy-associated HLH	Retrospective salvage cohort	15	Emapalumab as salvage therapy	Ferritin reduction; poor OS	Infections	[36]
	Malignancy-associated HLH	REAL-HLH, multicenter retrospective	17	Emapalumab-containing regimens, usually with other HLH- and malignancy-directed therapies	CXCL9 normalization/stability 87.5%; 12-month OS 22.1%	Infections; organ dysfunction	[37]
	Rheumatic disease-associated HLH/MAS	REAL-HLH, multicenter retrospective	15	Emapalumab-containing treatment	CXCL9 and ferritin reduction	Infection/viral reactivation risk	[38]
**HSCT**	Previously treated	Phase II–III, open-label	27	Emapalumab + dexamethasone before HSCT	HSCT 70%; post-HSCT survival 89.5%	Serious infections; infusion reactions	[8]
	Real-world	Multicenter retrospective	42	Emapalumab-containing therapy before HSCT	HSCT 73.8%; 12-month post-HSCT survival 73.7%	No discontinuations due to adverse events	[34]
	First HSCT after emapalumab	REAL-HLH transplant analysis	35	Emapalumab before and, in selected patients, during/post-conditioning	Engraftment 80%; 12-month post-HSCT OS 73.4%; mixed chimerism 37.1%	GVHD 54.3% (19/35); grade 3–4 acute GVHD in 5/13 acute cases	[39]
	Pre-HSCT emapalumab	Retrospective comparative cohort	50	Emapalumab within 21 days before conditioning	Mixed chimerism 48% vs. 77%; 5-year IFS 73% vs. 43%	No new emapalumab-specific safety signal identified	[40]
**MAS**	Still’s disease-associated Still’s disease-associated SLE-associated AOSD/SLE-associated refractory MAS	Phase II, open-label	14	Emapalumab + background therapy	MAS remission 92.9%; median remission 25 days	Viral infections; CMV events	[41]
		EMERALD, prospective open-label	25	Emapalumab-containing treatment	W8 ORR 64%; CR 44%	Infections/viral reactivation	NCT05001737
		EMERALD, prospective open-label	8	Emapalumab-containing treatment	W8 ORR 75%; CR 50%	Infections/viral reactivation	NCT05001737
		Retrospective cohort	9	Low-dose emapalumab	W8 ORR 88.9%; CR 88.9%	Infections during immunosuppression	[42]
**Ot** **her**	CAR T-cell-associated refractory CRS	Retrospective chart review	6	Emapalumab as salvage therapy	Clinical improvement 5/6	No major new signal reported	[43]
	CAR T-cell-associated high-grade CRS	Single-center retrospective	38	Emapalumab as salvage therapy	IFN-γ ↓96.9%; 6-month EFS 76.9%; OS 80.1%	No apparent short-term CART impairment	[44]
	HLH + high-risk TMA	Retrospective cohort	23	Concurrent IFN-γ and terminal complement blockade	TMA resolution 10/10; survival 10/10	Combination-therapy safety uncertain	[45]

Some patient populations may overlap across prospective studies, REAL-HLH analyses, and HSCT-focused cohorts. Abbreviations: pHLH, primary hemophagocytic lymphohistiocytosis; sHLH, secondary hemophagocytic lymphohistiocytosis; MAS, macrophage activation syndrome; HSCT, hematopoietic stem cell transplantation; GVHD, graft-versus-host disease; SLE, systemic lupus erythematosus; AOSD, adult-onset Still’s disease; CRS, cytokine release syndrome; TMA, thrombotic microangiopathy; EBV, Epstein–Barr virus; CMV, cytomegalovirus; CXCL9, C-X-C motif chemokine ligand 9; CR, complete response; ORR, overall response rate; OS, overall survival; EFS, event-free survival; IFS, intervention-free survival; W8, week 8.

## Data Availability

No new data were created or analyzed in this study. Data sharing is not applicable to this article.
